# Outcomes of Endodontic-Treated Teeth Obturated with Bioceramic Sealers in Combination with Warm Gutta-Percha Obturation Techniques: A Prospective Clinical Study

**DOI:** 10.3390/jcm12082867

**Published:** 2023-04-14

**Authors:** Denise Irene Karin Pontoriero, Edoardo Ferrari Cagidiaco, Valerio Maccagnola, Daniele Manfredini, Marco Ferrari

**Affiliations:** 1 Department of Prosthodontics and Dental Materials, University of Siena, 53100 Siena, Italy; 2 Department of Orthodontics, University of Padua, 35122 Padua, Italy

**Keywords:** clinical trial, endodontic outcomes, endodontically treated teeth, bioceramic sealers

## Abstract

The objective of this clinical study was to collect short-term endodontic outcomes of endodontic-treated teeth (ETT) obturated with different kinds of bioceramic sealers used in combination with warm gutta-percha obturation techniques. Methods: A total of 210 endodontic treatments in 168 patients were performed. At baseline, 155 sample teeth (73.8%) showed symptoms (tenderness or pain to percussion) and 125 (59.5%) showed periapical radiolucency. Periapical radiolucency was present in 125 cases (59.5%); of these, 79 showed a lesion of 5 mm or bigger (63.2%) while lower than 5 mm in 46 cases (36.8%). Regarding ETT with radiolucency, 105 of them (84%) were in coincidence with their need for retreatment and the other 20 (16%) were necrotic teeth. The obturation techniques that were used in this study were: the continuous wave of condensation technique in 75% of cases, and carrier-based technique in 25%. Four bioceramic sealers were used: CeraSeal in 115 cases, BioRoot in 35 cases, AH Plus Bio in 40 cases, and in 20 cases, BIO-C SEALER ION. Preoperative and recall radiographs of the roots were each assigned a periapical index (PAI) score by 2 blinded, independent, and calibrated examiners. The teeth were divided into outcome categories based on the following classification: healed, unhealed, and healing. The healed and healing categories were classified as success, and the unhealed category was classified as failure on the basis of loose criteria. Minimum follow-up period was 18 months. Results: The overall success rate was 99%, with 73.3% healed, 25.7% healing, and 0.95% not healed. The success rate was 100% for initial treatment and 98.2% for retreatment. Fifty-four (N = 54) teeth showed ongoing healing. All of them were retreatment cases with periapical lesions. Regarding the success (healed and healing) versus not healed, no significant difference was found between teeth with or without periapical lesions (*p* < 0.05). A statistically significant difference in the distribution of healed, healing, and not-healed teeth was found between the groups of teeth with baseline lesions < 5 mm and >5 mm in diameter (*p* < 0.01) and those with sealer groups (*p* < 0.01). The success rate of used bioceramic sealers was not statistically significant different (99.1%, 100%, 97.5% and 100%, respectively, for CeraSeal, BioRoot, AH Plus Bio, and BIO-C SEALER ION). Nonetheless, the distribution of healed, healing, and not-healed teeth was different between teeth sealed with different materials (*p* < 0.01). From the findings of this clinical study, the following conclusion can be drawn: a correct filling of root canals made with warm gutta-percha technique combined with a bioceramic sealer allows a high success rate in endodontically treated teeth.

## 1. Introduction

Bioceramic sealers (BS), also known as calcium silicate-based endodontic sealers, were introduced in dentistry and their mechanical, chemical, and biological properties were studied [1,2,3,4,5]. They were launched into the dental market and their popularity progressively increased among endodontists and dental practitioners. BS cements were first used to repair root perforation and in surgical endodontics as retro filling materials [6]. A fine formulation of these materials was made available and BS are now recognized as very useful in endodontic therapy. The BS are placed into the root canal using an easy technique and thanks to the filler size less than two microns, they can penetrate into the dentinal tubules sealing them. Additionally, BS can create a chemical bond with dental substrates and are sufficiently radiopaque [7,8,9] and have antibacterial properties [10,11,12]. Additionally, BS showed to be osteoinductive and biocompatible: these characteristics might help in bone regeneration of periapical lesions [13,14]. Because of their biocompatibility and intrinsic osteoinductive capacity, when an overfill happens, an inflammatory response will not take place and during hardening, when they come in contact with tissue fluids, calcium hydroxide reacts with phosphatase enzymes, resulting in the formation of hydroxyapatite [15]. Regarding their capacity to seal the apex, no significant differences were found in the quality of obturation when single-cone, warm condensation, and carrier-based techniques using bioceramic sealers were used [16,17]. Although the single cone technique needs a large amount of cement, and that can have voids and bubbles within the sealer itself, it was advocated as the main obturation technique in combination with BS [18]. Another aspect that supported combining the single cone technique with BS was that these materials should be used without the heat in order to not accelerate their setting [19,20]. Moreover, their hydraulic capability to penetrate into the dentinal tubules can enhance the retention of the sealer and create a mechanical barrier able to prevent bacteria leakage [7].

The long-term success of endodontic treatments is based on adequate 3-dimensional (3D) cleaning, shaping, and 3-dimensional obturation of the complex root canal system [21,22]. The role of endodontic sealers in combination with different types of endodontic obturation techniques was investigated and BS were proposed into the market as indicated only in combination with single-cone technique because the BS are unadvisable to come into contact with heat [19,23,24]. Otherwise, they can harden instantly. However, a recent study evaluated the use of several BS in combination with warm gutta-percha techniques, showing promising results [16].

Predictable and reliable results may be obtained only with clinical trials. Clinical trials are much more reliable than in laboratory studies made in both retrospective and prospective ways [25,26]. When a prospective clinical trial is made, only a few specific parameters are evaluated in a limited number of specimens and they take place in specialized centers. Through a retrospective study, a wider number of specimens can be collected and it may reflect more the clinical behavior of practitioners. The objective of this study was to evaluate outcomes of endodontically treated teeth (ETT) obturated with BS used in combination with warm gutta-percha obturation techniques.

The tested null hypotheses were: (1) there was no difference in the endodontic success of different BS; (2) there was no difference in the endodontic success of ETT with periapical lesions showing different sizes of the lesion (more or less than 5 mm); (3) there was no difference in the endodontic success of ETT with and without extrusion of BS; (4) there was no difference in the endodontic success of ETT of initial vs. retreated teeth.

## 2. Materials and Methods

Over 1 year (March 2020 to March 2021), one expert operator (DP) made 210 endodontic treatments in 168 patients (85 men, 83 women; age range: 19 to 81 years; media: 61 years). Patients required different endodontic therapies. Consecutive patients were selected from the authors’ offices. The size of the sample was calculated with a margin of error of 5%, and a confidence level of 95%, accordingly, with a population size of 500 of patients in need of endodontic treatment. Then, it was decided to include in this survey only primary endodontic-treated teeth or nonsurgical retreatments (112 of nonsurgical retreatments and 98 of primary endodontic treatments) with a follow-up of at least 18 months or longer (mean follow up 19.7 months) and all patients, after being endodontically treated, were placed in a recall periodical program of oral hygiene from the beginning of 2022.

The clinical protocol was performed in accordance with the 1964 Helsinki declaration and its later amendments or comparable ethical standards. All patients were informed and provided their written consent. The study was approved by the Ethical Committee of the University of Siena (protocol code PR001; data of approval 21 October 2019).

Inclusion criteria were the following: periodontally healthy or successfully treated patients in need of one or more endodontic treatments.

Exclusion criteria were the following: patients with an age lower than 18 years, pregnancy, disabilities, previous prosthodontic restorations of abutment teeth, deep bone defects, pulp capping, heavy occlusal contacts or history of bruxism, systemic disease or severe medical complications, allergic history concerning methacrylates, high incidence of caries, xerostomia, and lack of compliance.

A total of 210 teeth were collected and of them, 100 were maxillary posteriors (47.6%), 73 mandibular posteriors (34.7%), and 37 anterior teeth (17.7%), uppers and lowers; 85 ETT belonged to the mandible (40.5%) and 125 (59.5%) to maxillae.

At baseline, 155 sample teeth (73.8%) showed symptoms (tenderness/pain to percussion) and 125 (59.5%) had periapical radiolucency and of these, 79 showed a lesion of 5 mm or bigger (63.2%), while 46 showed a lesion smaller than 5 mm (36.8%). Regarding ETT with radiolucency, 105 of them (84%) were in need for retreatment and the other 20 (16%) were necrotic teeth.

The performed obturation techniques were the continuous wave of condensation technique in 158 cases (75%) and the carrier-based technique in 52 (25%), mainly in presence of very curved and narrow canals. All obturations were performed using a bioceramic sealer. Four BS were randomly selected accordingly with their availability: CeraSeal (Sweden & Martina, Due Carrare PD, Italy) in 115 cases (54.5%), BioRoot (Septodont, Saint Mour des Fousses, France) in 35 cases (16.7%), AH Plus Bio (Dentsply, Kostanz, Germany) in 40 cases (19%), and in 20 cases, (9.5%) BIO-C SEALER ION+ (Angelus, Londrina, Brasil).

Table 1 reports demographic characteristics of the patient.

The following preoperative data were recorded for each case: demographic data, tooth location, number of root canals, previous endodontic treatment, clinical signs and symptoms, vitality tests, and radiographic periapical status. Based on these findings, the preoperative condition was classified as one of the following: vital, non-vital, previously endodontically treated, with or without periapical lesion, and symptomatic or asymptomatic.

For each tooth, the following intra-operative data were written in the clinical records: how many appointments were needed to complete the treatment, presence of complications such as perforation, breakage of files and flare-up; length of canal filling (at apical level, 1 mm short or more and beyond). The endodontic and restorative procedures were performed accordingly with Pontoriero et al. [27]. Finally, the roots were obturated with gutta-percha cones and one of the four bioceramic sealers tested following a continuous wave of condensation technique (75%) or a carrier-based technique (Thermafil, Dentsply, Konstanz, Germany) depending on the root canal anatomy.

The build-up and restorative procedures were performed as described by Pontoriero et al. [27].

Postoperatively, the same preoperative data were collected also accordingly with Pontoriero et al. [27] and the evaluation parameters made by the European Society of Endodontology 2006 [28] were followed. The primary authors (DP) made all the visits at the follow-ups.

The entity of the lesion was recorded and evaluated. Consequently, endodontic treatments were classified as failures when pain was present, and/or swelling and sinus tract. Radiographically, a lesion appeared after endodontic treatment, when a pre-existing lesion increased in size, and when a lesion remained the same, it was considered as failure [29]. Additionally, preoperatively and at each recall, the Periapical Index (PAI) score system was used [30,31] by 2 blinded, independent, and calibrated examiners (D. P., M.F.) and each endodontically treated tooth received the highest score for any of the roots.

The treated roots were classified as the following [32]:

1. Healed: teeth in good function, without symptoms and without radiographic periapical lesion;
2. Not healed: nonfunctional teeth with symptoms with or without radiographic periapical lesion or teeth without symptoms with unchanged, new, or enlarged radiographic periapical lesion;
3. Healing: teeth that are without symptoms and good function, with a decreased size of radiographic periapical lesion.

**Figure 1:**
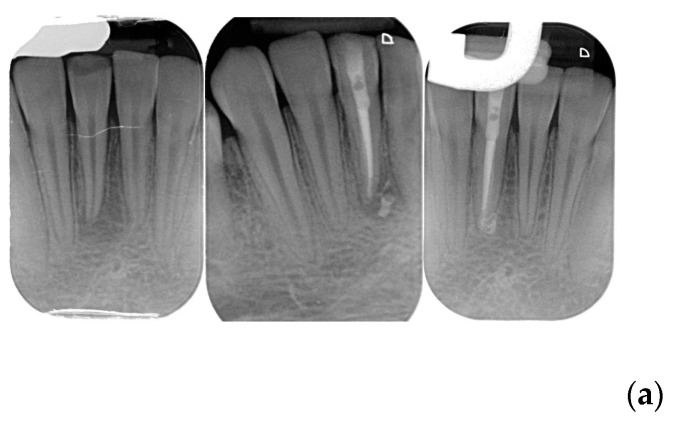

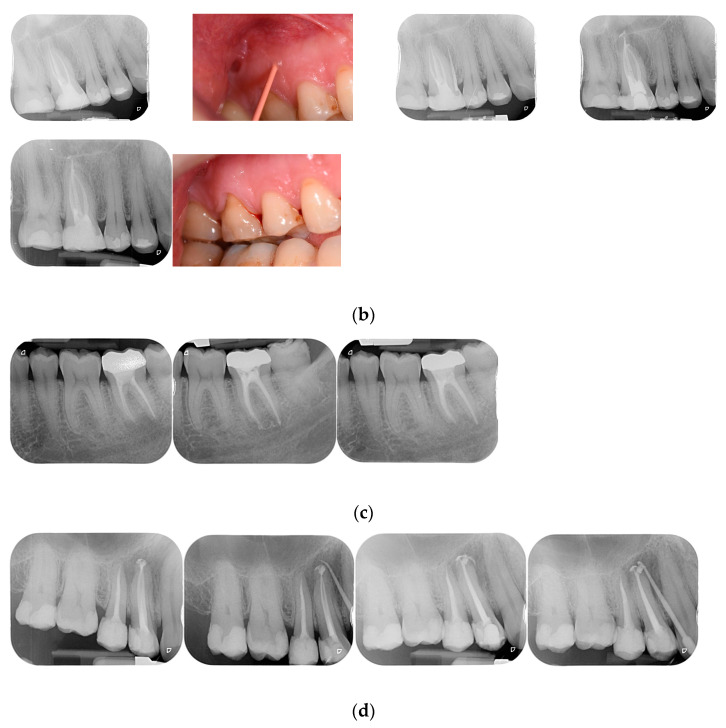
(**a**) Healed lower incisor at 12 months recall. (**b**) Healed upper first molar at 18 months recall. (**c**) A lower second molar in healing process after 6 months. (**d**) Not healed first upper bicuspid at 8 months recall.

The clinical evaluation ‘success’ was referred to healed and healing categories and ‘failure’ to the not-healed teeth was classified as failure. Figure 1 show examples of each category. When a disagreement on the radiographic and/or clinical evaluation between the two evaluators was present, a discussion was made and a final consensus was reached. Examples of each outcome category are shown in Figure 1a–d.

### 2.1. Outcome Evaluation

The outcomes assessment are reported in Table 2 and classified accordingly with Chybowski et al., 2018 [32]. In order to identify possible prognostic factors, many variables related to the patient, the tooth, and the treatment were evaluated. Patient factors examined included the sex and age of the patient. Tooth-related factors included tooth type, pulpal and periapical diagnosis, pocket depths, sinus tract, presence/absence of periapical lesion, lesion size, and preoperative percussion and palpation sensitivity. Treatment factors evaluated included treatment type (initial treatment or retreatment), type of BS, sealer extrusion, follow-up time.

### 2.2. Statistical Analysis

For the purpose of statistical analysis, contingency tables were created with the success outcome categories in column (i.e., number of healed, healing, not-healed teeth) and the parameters of potential clinical interest in row (i.e., number of teeth in patients with age > 50 years, with baseline size of the lesion > 5mm, with apical extrusion of sealer, treated with different sealers). The expected percentage of successful treatments, arbitrarily set at 98%, was used as the basis to calculate the needed sample size to identify a 2% between-group difference with the conventional 5% type I error and 20% type II error. Based on that, 77 teeth per group were needed to have an 80% statistical power [33]. The Pearson chi-square test was used to compare the distribution of values in the different cells. A *p* value < 0.05 was considered significant, and all tests were 2-sided. Statistical analysis was performed with SPSS v26.0 software (IBM Corp, Armonk, NY, USA).

## 3. Results

The overall success rate was 99%, with 73.3% healed, 25.7% healing, and 0.95% not healed. The success rate was 100% for initial treatment and 98.2% for retreatment. Fifty-four (N = 54) teeth showed ongoing healing. All of them were retreatment cases with periapical lesions. Regarding the success (healed and healing) versus not healed, no significant difference was found between teeth with or without periapical lesions. However, 154 ETT were classified as healed and 54 ETT as healing.

Patients younger or older than 50 years had a similar rate of healed teeth (75.8% vs. 69.5%), without any difference in success rate (98.8% vs. 99.2%) (*p* = 0.31).

A statistically significant difference in the distribution of healed, healing, and not-healed teeth was found between the groups of teeth with baseline lesions < 5 mm and >5 mm in diameter (*p* < 0.01), showing that when the lesions were smaller, the healing process was faster than those with lesions that were bigger than 5 mm in diameter.

Eighty-five treated teeth (40.5%) showed extrusion of the sealer on one or more root(s). The distribution of healed, healing, and not-healed teeth was different between teeth with or without sealer extrusion, with the latter group including a higher percentage of healed teeth (*p* < 0.01). In particular, the two cases recording an outcome “not healed” showed extrusion of the sealer, with a periapical lesion wider than 5 mm that required retreatment. The presence of extrusion of the sealer was more frequently observed when a preoperative lesion was present (69%) compared with when no lesion was present (31%) (*p* < 0.01). Although success was not different between initial treatment and retreatment groups, and between groups with and without sealer extrusion, the number of healed roots was higher on roots of initial treatment and without sealer extrusion at the apex.

The success rate of used BS was not statistically significantly different (99.1%, 100%, 97.5%, and 100%, respectively, for CeraSeal, BioRoot, AH Plus Bio, and BIO-C SEALER ION). Nonetheless, the distribution of healed, healing, and not-healed teeth was different between teeth sealed with different materials (*p* < 0.01).

After being endodontically treated, 125 (59.5%) ETT were restored by direct resin composite restorations using mainly a fiber reinforced flowable resin composite (EveryXFlow GC Co., Tokyo, Japan), and 85 (40.5%) posts were luted. A total of 50 (23.8%) direct restorations remained as final restoration, 92 single crowns (43.8%), 30 (14.3%) partial adhesive crowns, and 38 (18.1%) abutments of fixed bridges were the final treatments.

## 4. Discussion

Recently, BS were used in clinical trials under controlled conditions. Some authors highlighted that there were no differences between BS and resin and/or zinc phosphate sealers [34,35,36,37,38,39,40], which was also in case of unintentional apical extrusion of sealers [40]. The BS were used with single cone obturation technique [32,34,35,36,37,38,39,40,41] versus zinc phosphate or resin sealers in combination with warm vertical compaction, and the clinical results were always very good. From the results of these clinical trials, it can be speculated that BS can be used in combination with single cone as continuous wave of condensation techniques, and their outcomes are similar to those observed with zinc phosphate and/or resin endodontic sealers.

In this clinical study, success (healed and healing), and failure (not healed) rates were, respectively, 99% and 0.95%. Healed was recorded in 100% of first treatment cases, whilst in case of retreatment in 55.2% of cases were classified as healed, and 43.2% were still healing; this can be due to the fact that almost all retreatment cases showed a periapical lesion and they needed a longer time to heal completely. When there was no periapical lesion, 100% success was recorded.

Four types of BS were tested, and their success rates were **s**imilar: between 97.5% and 100%. For that, the first null hypothesis was accepted. However, it must be highlighted that only one material was used in more than 100 cases (CeraSeal in 115 sample teeth), whilst BioRoot, AH Plus Bio, and BIO-C SEALER ION were used, respectively, in 35, 40, and 20 teeth. A wider number of samples of the last three BS and a more uniform distribution of them are desirable in further randomized controlled trials.

Regarding the second tested null hypothesis, i.e., that there was no difference in the endodontic success of ETT with periapical lesion of more or less 5 mm in size at the beginning of the treatment was accepted, the cumulative success rate (healed and healing) showed no statistical significance difference.

However, the size of the periapical lesion showed to be important; when the lesion was lower than 5 mm in diameter, 81% of roots were classified as healed, but when the lesion was wider than 5 mm, only 54.3% were healed. These results were expected because of the short-term observation time.

The third tested null hypothesis was that there was no difference in the endodontic success of ETT with and without extrusion was accepted. In fact, the cumulative success rate (healed and healing) showed, respectively, 97.6% and 100%.

However, when there was no extrusion of the sealer, only 8.8% of ETT showed healing, whilst when it was present, 50.6% of roots were classified as healing. It was also noted that extrusion was usually present when the apex was already opened by the necrosis and was combined with the periapical lesion. From a clinical point of view, it was observed that the presence of postoperative pain was not influenced by the sealer’s extrusion [26].

Regarding the fourth null hypothesis that there was no difference in the endodontic success of ETT of initial vs. retreated teeth, it was accepted because there was no difference in cumulative success rate between the two groups.

Only two failures were recorded and were both retreatments, with a periapical lesion, wider 5 mm in size, with sealer extrusion. From the other side, 208 (99%) cases were classified as a success, and these excellent results can be due to the appropriate shaping and cleaning of root canals [32,41], the obturation procedure [32], the hydraulic effect that pushes the bioceramic sealer into the dental tubules sealing them [42,43,44], and the osteogenic characteristic of this new material [45].

When all the roots were obturated using warm techniques, voids were never noted within the obturation. Additionally, no one root showed short obturation in length.

The clinical evaluation of endodontic outcomes that consider “success” the complete resolution of the periapical radiolucency can be “strict” [46] or “stringent” [47], while choosing a mere reduction in the size of the periapical radiolucency [29,32] was described as setting a “loose” [47] or “lenient” [46] threshold. In this study, it was decided to follow a “loose” [34] or “lenient” [35] threshold. In order to support the adoption of “loose” criteria, the radiographic assessment method was chosen [31]. This system provided a scale of five scores, ranging from healthy to severe periodontitis with exacerbating features [31]. It was based on radiographs with verified histological diagnosis and can be suitable in epidemiological studies [48]. Additionally, the endodontic failure usually occurs within the first years of clinical service [49]. However, it must be noted that the observation time was short, too short to permit complete healing of wide periapical radiolucency [28,48]. However, the expected success rates using the “strict” criteria would be lower than those based on the “loose” criteria [28,29]. All the patients collected in this study were in a recall program to confirm, or disprove, the outcomes under a longer observation period.

Comparing the outcomes of this study with those recently published by the same authors [27], it may be noted that the skill and knowledge of the operators can allow high quality of endodontic treatment and good prognosis. The “operator” could be considered one of the most important factors concerning the outcomes in dentistry and in endodontics.

Regarding the type of build-up, the findings of this study confirmed that the materials and procedure used do not affect the final outcome [50].

Some limitations of this study can be underlined. Firstly, the wider number of ETT should be enrolled; also, the good outcomes of this study were related to the skill and knowledge of one single expert, and it would be of some interest to extend the number of endodontists. Additionally, the limited observation time was short and the patients of this study were in a recall program to collect longer data and to confirm the reported outcomes. Finally, a multicenter prospective study is desirable to confirm the findings of this study.

## 5. Conclusions

From the findings of this clinical study, the following conclusion can be drawn: a proper obturation of root canals made with warm gutta-percha technique combined with a bioceramic sealer allows a high success rate in endodontically treated teeth.

A periapical lesion does not compromise the quality of the final outcomes.

## Figures and Tables

**Table 1:** Population demographics and type of treatment. (PARL, periapical radiolucency; RCT, root canal treatment; ReTx, retreatment).

Sex (*n =* 168)	Male 85 (50.6%)	Female 83 (49.4%)		
Age	**>**50 63 (37.5%)	<50 105 (62.5%)		
Type of Treatment (*n =* 210)	Initial RCT 98 (46.7%)	ReTx 112 (53.3%)		
Tooth Type (*n =* 210)	Maxillary Anterior 15	Maxillary Posterior 110	Mandibular Anterior 12	Mandibular Posterior 73
PARL Presence	Present 125 (59.5%)	Absent 85 (40.5%)		
Lesion Size (*n =* 125)	>5 mm 46 (36.8%)	<5 mm 79 (63.2%)		
Bioceramic Sealers (*n =* 210)	CeraSeal 115 (54.5%)	BioRoot 35 (16.7%)	AH Plus Bio 40 (19%)	Bio-C SEALER ION 20 (9.5%)

**Table 2:** The table reports the full recorded outcomes.

Factors/Demography		Healed	Healing	Not Healed	Success	*p* Value
	Total (*n* = 210)	154 (73.3%)	54	2	208	
			−25.70%	−0.95%	−99%	
**Age (years)**						0.31
**(*n =* 168)**						
	>50 years					
	(*n* = 82) (39%)	57 (69.5%)	24 (29.3%)	1 (1.2%)	81 (98.8%)	
	<50 years					
	(*n* = 128) (61%)	97 (75.8%)	30 (23.4%)	1 (0.8%)	127 (99.2%)	
**Treatment type**						Not applicable
**(*n =* 210)**						
	Initial					
	(*n* = 98) (46.7%)	98 (100%)			98 (100%)	
	ReTx					
	(*n* = 112) (53.3%)	56 (50%)	54 (48.2%)	2 (1.8%)	110 (98.2%)	
**Lesion**						Not applicable
**(*n =* 210)**						
	Present					
	(*n* = 125) (59.5%)	69 (55.2%)	54 (43.2%)	2 (1.6%)	123 (98.4%)	
	Absent					
	(*n* = 85) (40.5%)	85 (100%)			85 (100%)	
**Lesion Size**						<0.01
**(*n =* 125)**						
	>5 mm					
	(*n* = 46) (36.8%)	25 (54.3%)	19 (41.3%)	2 (4.4%)	44 (95.6%)	
	<5 mm					
	(*n* = 79) (63.2%)	68 (86%)	11 (13.9%)		79 (100%)	
**Sealer Extrusion**						<0.01
**(*n =* 210)**						
	Present					
	85 (40.5%)	40 (47%)	43 (50.6%)	2 (2.4%)	83 (97.6%)	
	Absent					
	125 (59.5%)	114 (91.2%)	11 (8.8%)		125 (100%)	
**Bioceramic Sealers**						<0.01
**(*n =* 210)**						
	CeraSeal					
	(*n* = 115) (54.8%)	96 (83.5%)	18 (15.6%)	1 (0.9%)	114 (99.1%)	
	BioRoot					
	(*n* = 35) (16.7%)	17 (48.6%)	18 (51.4%)		35 (100%)	
	AH Plus Bio					
	(*n* = 40) (19%)	28 (70%)	11 (27.5%)	1 (2.5%)	39 (97.5%)	
	BIO-C SEALER ION					
	(*n* = 20) (9.5%)	13 (65%)	7 (35%)		20 (100%)	

## Data Availability

The data presented in this study are available on request from the corresponding author. The data are not publicly available due to containing personal information.
